# Contrasting Effects of Histone Deacetylase Inhibitors on Reward and Aversive Olfactory Memories in the Honey Bee

**DOI:** 10.3390/insects5020377

**Published:** 2014-06-10

**Authors:** Gabrielle A Lockett, Fiona Wilkes, Paul Helliwell, Ryszard Maleszka

**Affiliations:** Research School of Biology, The Australian national University, Canberra ACT 0200, Australia; E-Mails: G.A.Lockett@soton.ac.uk (G.A.L.); Fiona.wilkes@anu.edu.au (F.W.); Paul.helliwell@anu.edu.au (P.H.)

**Keywords:** histone acetylation, epigenetics, honey bee, brain plasticity, aversive memory, reward memory

## Abstract

Much of what we have learnt from rodent models about the essential role of epigenetic processes in brain plasticity has made use of aversive learning, yet the role of histone acetylation in aversive memory in the honey bee, a popular invertebrate model for both memory and epigenetics, was previously unknown. We examined the effects of histone deacetylase (HDAC) inhibition on both aversive and reward olfactory associative learning in a discrimination proboscis extension reflex (PER) assay. We report that treatment with the HDAC inhibitors APHA compound 8 (C8), phenylbutyrate (PB) or sodium butyrate (NaB) impaired discrimination memory due to impairment of aversive memory in a dose-dependent manner, while simultaneously having no effect on reward memory. Treatment with C8 1 h before training, 1 h after training or 1 h before testing, impaired aversive but not reward memory at test. C8 treatment 1 h before training also improved aversive but not reward learning during training. PB treatment only impaired aversive memory at test when administered 1 h after training, suggesting an effect on memory consolidation specifically. Specific impairment of aversive memory (but not reward memory) by HDAC inhibiting compounds was robust, reproducible, occurred following treatment with three drugs targeting the same mechanism, and is likely to be genuinely due to alterations to memory as sucrose sensitivity and locomotion were unaffected by HDAC inhibitor treatment. This pharmacological dissection of memory highlights the involvement of histone acetylation in aversive memory in the honey bee, and expands our knowledge of epigenetic control of neural plasticity in invertebrates.

## 1. Introduction

Epigenetic control systems such as DNA methylation and post-translational histone modifications have the capacity to control the organism’s gene expression potential without changing the underlying DNA sequence. Over the past decade these long-lasting yet flexible mechanisms have been shown to be critical for behavioural memory (reviewed by [1,2]) along with other processes including disease [2,3]. We previously showed that DNA methylation is involved in memory processing in the honey bee [4] suggesting that the function of this epigenetic process in neural plasticity is phylogenetically conserved [2,5,6]. Regulation of chromatin structure through post-translational modification of histone proteins, primarily histone H3 phosphorylation and acetylation, is an important early step in the induction of synaptic plasticity and formation of long-term memory.

Histone acetylation levels are maintained in a dynamic equilibrium by histone deacetylase (HDAC) and histone acetyltransferase (HAT) enzymes. Histone acetylation was first shown to have a role in memory in *Aplysia* [7], and training in the crab alters histone acetylation in the central brain [8]. Treatment with HDAC inhibitors increases levels of acetylation of histone tails which opens up chromatin structure facilitating gene expression [9]. HDAC inhibition improves many types of memory in rodents, including contextual fear conditioning [10,11,12], extinction of fear [12,13], fear potentiated startle [14], novel object recognition [15,16], novel taste learning [17], eye blink classical conditioning [16] and performance in the Morris water maze spatial memory test [18]. HDAC inhibition also strengthens context-signal memory after weak training in the crab [8]. However, HDAC inhibition has also been shown to impair novel object recognition in the rat [19]. Different types of memory can be differentially affected by changes to histone acetylation machinery: in mouse *cbp*^+/−^ mutants, novel object recognition and fear learning are impaired yet spatial navigation is unaffected [20].

Recently, Merschbaecher and colleagues demonstrated the role of histone acetylation in olfactory associative reward memory in the honey bee [21]. They showed that learning induces changes to histone H3K9 and H3K18 acetylation in the honey bee central brain—which, since this is the same part of the brain in which *Dnmt3* is upregulated following training [4], suggests that the interplay between DNA methylation and histone acetylation observed in rodent memory [22] also occurs in honey bee memory. Their experiments utilised an olfactory associative memory paradigm to demonstrate that HDAC inhibition with the drug trichostatin A (TSA) improves reward memory, and that this lasts longer with stronger training.

Aversive stimuli are frequently used in classical conditioning and operant conditioning studies in range of invertebrate species. Adult forager honeybees have been shown to be capable of learning to withhold the proboscis extension reflex (PER) when presented with an odour and sugar solution coupled with an electric shock [23], and olfactory association can also train them to extend their sting [24]. Many studies have examined the role of histone acetylation in aversive memory in other animals [8,11,12], yet to our knowledge the involvement of histone acetylation in aversive memory in the honey bee has not been examined.

The honey bee is a popular invertebrate model in behavioural and molecular studies, including epigenetic analyses [25,26,27,28]. The recent characterisation of histone post-translational modifications in the honey bee [29], in conjunction with its popularity for studies of memory [30] improves the utility of this model animal for epigenetic dissection of memory. We used a modified version of the PER assay [31,32,33] which measures discrimination learning, in conjunction with treatment with HDAC inhibiting drugs. We selected the HDAC inhibitors APHA compound 8 (C8), phenylbutyrate (PB) and sodium butyrate (NaB) to assess the role of histone acetylation in aversive memory in the honey bee. C8 belongs to the 3-(4-aroyl-1*H*-2-pyrrolyl)-*N*-hydroxy-2-propenamides (APHAs), a class of recently developed hydroxamic acid type HDAC inhibitors [34,35] of which C8 has the most potent HDAC inhibiting activity [36]. PB and NaB are short-chain fatty acid HDAC inhibitors. PB ameliorates memory deficits in rodent models [37], and NaB has been the HDAC inhibitor of choice in many recent neuroepigenomic studies, where its ability to increase histone acetylation levels has been confirmed *in vivo* (e.g., [18]).

## 2. Experimental Section

### 2.1. Olfactory Conditioning Assays

Individual frames of brood comb were removed from an experimental hive, transferred to an incubator and kept at a constant 32 °C, ~80% humidity. Bees were collected on their day of emergence and kept in groups of 50–100 in mesh cages until they reached six days of age. Age-matched bees were used to reduce variability unrelated to the experiment, and bees demonstrate consistent capacity for learning at the age used in our assay [4,38]. Our olfactory associative learning procedure [32] was based on that of Bitterman *et al.* [31]. Six day-old individual bees were anaesthetised on ice until barely moving, then secured in thin-walled aluminium tubes (7 mm diameter) using strips of fabric-reinforced tape, leaving the head and antennae free to move while also leaving the dorsum of the thorax exposed. Bees were fed on 1 M sucrose solution once per day, and left overnight at 25 °C. Any bee failing to respond was discarded before training.

Learning and memory were assessed using a PER olfactory association paradigm in which bees are required to discriminate between rewarding (CS+) and aversive (CS−) odours as conditioned stimuli. In this PER paradigm, discrimination learning typically increases during training and is quite robust in reaching ~66% at the 24 h retention test (e.g., [4,32]). The CS+ was limonene (4 μL/mL) and the CS− was natural vanilla (4 μL/mL), and these were associated with 1 M sucrose and saturated NaCl solution unconditioned simuli (USs) respectively [4,32]. The bee was first allowed to smell the CS for 5 s, then one antenna was touched with the US solution, leading to the extension of the proboscis and the tasting of the sucrose or saline solution. At 7 d of age, bees were given acquisition training consisting of 3 double (*i.e.*, both CS+ and CS−) training trials at 6 min inter-trial intervals [39]. Exactly 24 h after training bees were tested for the correct double CS/US association, with performance in the discrimination task (PER %) expressed as the proportion of bees making the correct double choice out of all bees making a choice [32]. When CS+ and CS− responses were analysed separately, the results for each CS are presented as the percentage correct conditioned response to each, *i.e.*, percentage proboscis extension for CS+ and percentage withholding the proboscis for CS−. A small exhaust fan positioned behind the bees was employed throughout the duration of the experiment.

Animals were tested for memory 24 h after training, by presenting both CSs to each bee and noting the presence or absence of proboscis extension. During testing the order of CS presentation was deliberately reversed in relation to the training procedure, to rule out the possibility that bees may have simply learnt the order of stimulus presentation. For each experiment 2–5 independent biological replicates were performed.

Bees were treated with the HDAC inhibitors APHA compound 8 (C8; Sigma-Aldrich, St. Louis, MO, USA) or PB (Sigma-Aldrich) delivered topically to the dorsal thorax dissolved in dimethylsulfoxide (DMSO) or dimethylformamide (DMF), or NaB (Sigma-Aldrich) in ringer solution [40] injected into the thoracic scutellar fissure as described previously [32]. Controls were treated with vehicle only. Injection is a popular application method for honey bees [32], though not an option for water-insoluble drugs such as C8. In honey bees, topical application of water-insoluble compounds dissolved in DMF to the thorax is at least as effective as thoracic injection in terms of amount reaching the brain [41]. The treatment volume used was always 1 µL and the dose was varied by altering the concentration of HDAC inhibitor. HDAC inhibitors were administered 1 h before training, 1 h after training or 1 h before testing as described for each experiment. Survival rates across all experiments described herein were not significantly different between controls and bees treated with C8, PB or NaB (*p* > 0.05, χ^2^ test).

### 2.2. Sucrose Sensitivity Assays

Newly emerged bees were obtained as above, and tested for sucrose sensitivity at 6, 7, 8 or 9 d of age using previously described methods [42]. For each experiment 2–3 independent biological replicates were performed. Briefly, on the morning of the day of testing bees were restrained in tubes and allowed to adjust for a total of 4 h in a humid incubator; no food was available during this time. 1 h before testing each bee was treated with 1 µL of 10 mM C8 or PB, or vehicle only. To test sucrose sensitivity each bee was stimulated alternately with water and increasingly sweet solutions by touching a drop briefly to the right antenna. Any bees extending their proboscis to water more than twice were regarded as sensitised and not included in calculations of group average sucrose sensitivity. Bees which did not extend their proboscis to any solution were also discarded. Biological replicates of each experiment were pooled and the average indices of each experimental group were compared.

### 2.3. Locomotor Activity Assays

Newly emerged bees were obtained as described above, and tested for locomotor activity at 7 or 8 d of age. Assay design was based largely on previously described methods [43,44]. Two independent biological replicates of the 7 d and 8 d experiments were performed, and one of the caffeine positive control. Bees were chilled briefly on ice, and then treated with 1 µL of 10 mM C8, 10 mM PB, 100 mM caffeine or vehicle only. Bees were then kept in a dark humid incubator for 1 h, then tested for locomotor activity on a sheet of base wax held vertically in a frame box. Locomotor activity was assessed by placing each bee on the wax, and covering it with the lid of a plastic Petri dish (Sarstedt AG & Co., Nümbrecht, Germany) (diameter 147 mm, depth 8 mm).

Each bee was given 1 min to adjust after which the number of line crossings (the number of times the bee’s head crossed a line) and instances of head cleaning were counted over a 4 min period. Each bee was tested using a fresh Petri dish lid, and dishes were washed between experiments.

### 2.4. Statistical Analyses

PER data were analysed for statistical significance using the χ^2^ test where all values in the χ^2^ expected values matrix were greater than 5, otherwise Fisher’s exact tests were used.

Sucrose sensitivity indices were assessed for statistically significant differences using Mann-Whitney *U* tests. Locomotor activity data (number of line crossings per minute and number of instances of head cleaning per minute) were tested for normality (*p* > 0.05, Kolmogorov-Smirnov tests) and where data were normally distributed *t* tests were used to determine statistical significance, otherwise non-parametric Mann-Whitney *U* tests were used. All tests were two-tailed. Survival rates were compared between HDAC inhibitor treatment and control groups using χ^2^ tests (all values in χ^2^ expected values matrix were greater than 5).

## 3. Results

### 3.1. C8 and PB Impair Aversive Memory but not Reward Memory in a Dose-Dependent Manner

**Figure 1:**
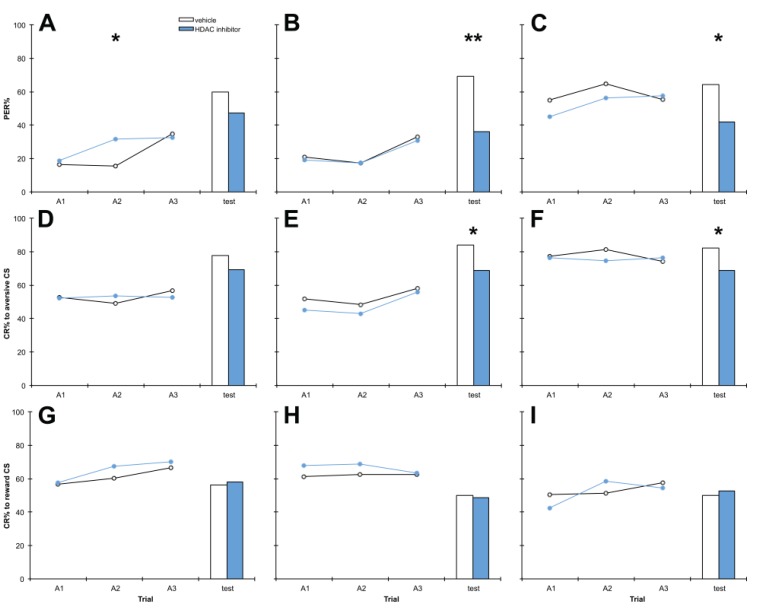
The histone deacetylase (HDAC) inhibitor compound 8 (C8) impairs aversive memory at test at doses 10 mM or greater. Bees were treated with 1 mM (**A**, **D**, **G**), 10 mM (**B**, **E**, **H**) or 20 mM (**C**, **F**, **I**) C8 or vehicle only, 1 h after training. Panels **A**–**C** show discrimination learning and memory, panels **D**–**F** show aversive learning and memory and panels **G**–**I** show reward learning and memory, during acquisition training (A1,A2,A3) and at the retention test. 1 mM: *n* C8 = 124, *n* vehicle = 89; 10 mM: *n* C8 = 80, *n* vehicle = 76; 20 mM *n* C8 = 80, *n* vehicle = 90. * *p* < 0.05, ** *p* < 0.01.

To first establish toxicity and optimal doses for the HDAC inhibitors APHA compound 8 (C8) and phenylbutyrate (PB) in PER assays, we treated bees with a range of doses of each drug. Bees were treated with 1 mM, 10 mM or 20 mM C8, or vehicle only, 1 h after training (Figure 1). Treatment with 10 mM or 20 mM C8 significantly impaired discrimination memory at the test 24 h after training (*p* = 0.003, 0.034 respectively). When aversive and reward responses are separated, it can be seen that aversive memory was significantly impaired at these doses (*p* = 0.023, 0.040 respectively). Treatment with 1 mM C8 was insufficient to produce this effect (*p* = 0.186). No significant effects on reward learning or memory were observed at any dose (*p* > 0.05). In the 1 mM experiment, by chance bees in the HDAC inhibitor treatment group performed better at the second training trial than control bees, however this seems to have had no consequence on performance at test. This effect was visible when discrimination learning was examined, but disappeared when results were separated into aversive and reward.

Survival rates of C8-treated bees were not significantly different from controls at 1 mM, 10 mM or 20 mM (*p* > 0.05). As 10 mM C8 was the lowest dose that produced an effect on memory, this dose was selected for subsequent experiments.

**Figure 2:**
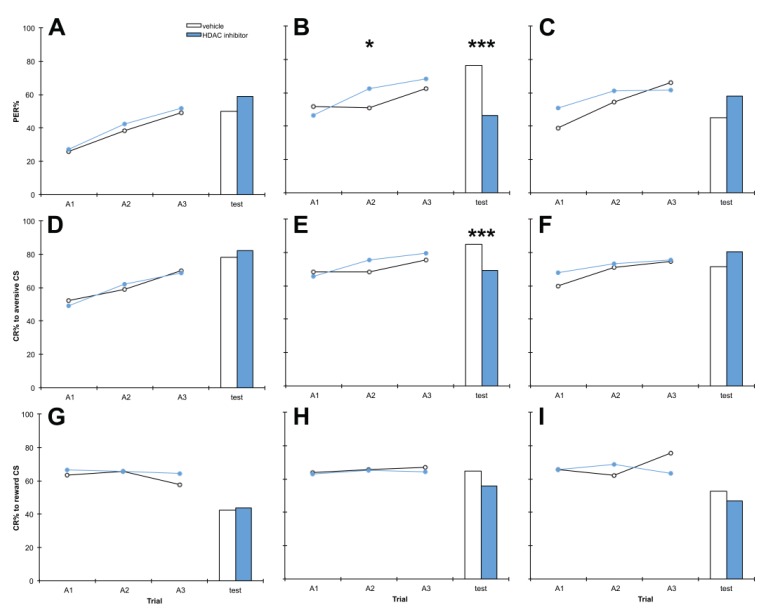
The HDAC inhibitor phenylbutyrate PB) impairs aversive memory at test at 10 mM but not 1 mM or 50 mM. Bees were treated with 1 mM (**A**, **D**, **G**), 10 mM (**B**, **E**, **H**) or 50 mM (**C**, **F**, **I**) C8 or vehicle only, 1 h after training. Panels **A**–**C** show discrimination learning and memory, panels **D**–**F** show aversive learning and memory and panels **G**–**I** show reward learning and memory, during acquisition training (A1,A2,A3) and at the retention test. 1 mM: *n* PB = 90, *n* vehicle = 90; 10 mM: *n* PB = 227, *n* vehicle = 227; 50 mM *n* PB = 90, *n* vehicle = 90. * *p* < 0.05, *** *p* < 0.001.

Similarly, we treated bees with 1 mM, 10 mM or 50 mM PB, or vehicle only, 1 h after training (Figure 2). 10 mM PB treatment impaired discrimination memory at test (*p* = 0.000), again this appears to be due to significant impairment of aversive memory at test (*p* = 0.000), with PB treatment having no effect on reward memory at test. Memory impairment by PB was detected at 10 mM PB, but not 1 mM or 50 mM (*p* = 0.547, 0.183 respectively). No significant effects on reward learning or memory were observed at any dose (*p* > 0.05). In the 10 mM experiment, by chance bees in the PB treatment group performed better during the second training trial than control bees for discrimination learning (*p* = 0.048), however in the test there was a highly significant difference in the opposite direction (*p* = 0.000), therefore this chance difference does not seem to have had any lasting effect.

Survival rates of PB-treated bees were not significantly different from controls at 1 mM, 10 mM or 50 mM (*p* > 0.05). As 10 mM PB was the dose that produced the observed effect on aversive memory, this dose was selected for subsequent experiments.

### 3.2. Treatment with C8 1 h before Training, 1 h after Training or 1 h before Testing Impairs Aversive Memory but Has no Effect on Reward Memory

**Figure 3:**
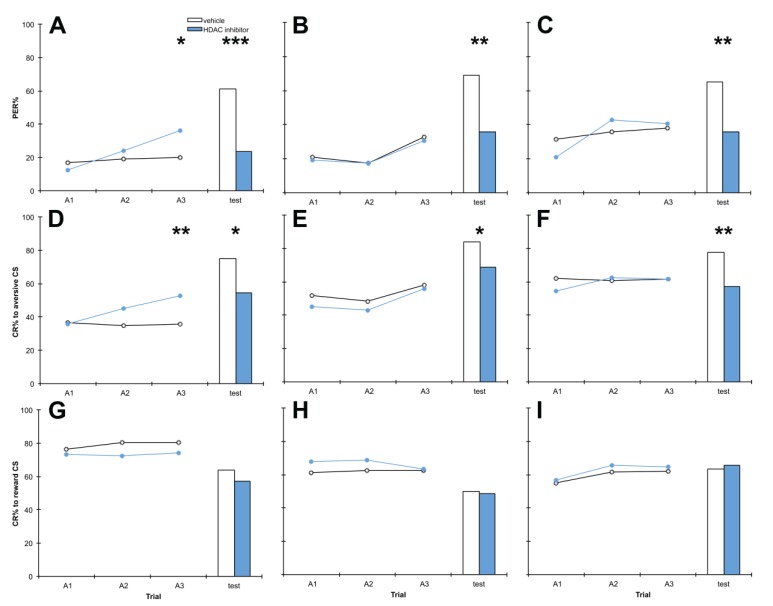
The HDAC inhibitor C8 impairs aversive memory but has no effect on reward memory. Bees were treated with 10 mM C8 or vehicle only, 1 h before training (**A**, **D**, **G**), 1 h after training (**B**, **E**, **H**); also shown in Figure 1, included here for comparison), or 1 h before testing (**C**, **F**, **I**). Panels **A**–**C** show discrimination learning and memory, panels **D**–**F** show aversive learning and memory and panels **G**–**I** show reward learning and memory, during acquisition training (A1,A2,A3) and at the retention test. 1 h before training: *n* C8 = 79, *n* vehicle = 64; 1 h after training: *n* C8 = 80, *n* vehicle = 76; 1 h before testing *n* C8 = 79, *n* vehicle = 72. * *p* < 0.05, ** *p* < 0.01, *** *p* < 0.001.

To better dissect the effects of HDAC inhibitor treatment on memory, bees were treated with C8 or vehicle 1 h before training, 1 h after training or 1 h before testing. Our results showed that C8 treatment 1 h before training, 1 h after training or 1 h before testing impaired discrimination memory at test, relative to vehicle-treated controls (*p* = 0.000, 0.003, 0.001 respectively) (Figure 3). As in the previous two experiments, this impairment of discrimination memory appears to be attributable to impairment of aversive memory at test, where significant differences were observed (*p* = 0.011, 0.023, 0.003 respectively). Treatment 1 h before training also significantly enhanced discrimination learning in the third training session (*p* = 0.016), due to an improvement in aversive learning (*p* = 0.009) without any change in reward learning (*p* = 0.243). Reward learning and memory were unaffected by C8 treatment at all three treatment times (*p* > 0.05).

### 3.3. Treatment with PB 1 h after Training Impairs Aversive Memory but Has no Effect on Reward Memory

**Figure 4:**
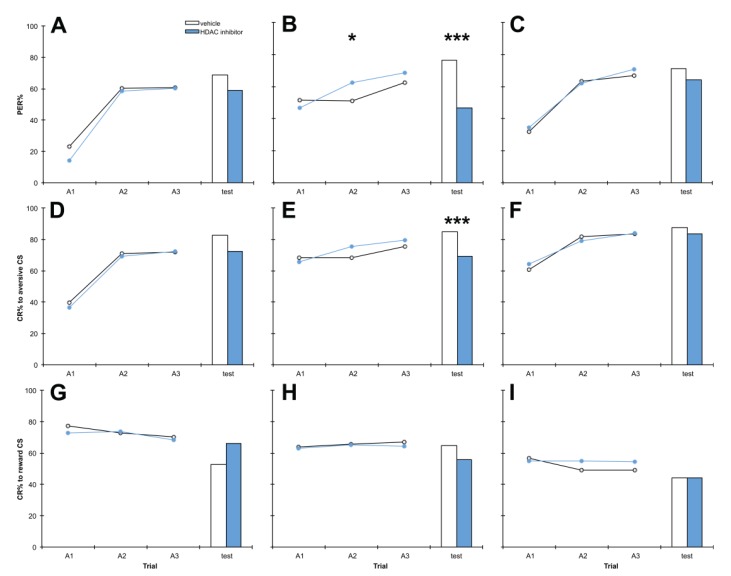
The HDAC inhibitor PB impairs aversive memory but has no effect on reward memory. Bees were treated with 10 mM PB or vehicle only, 1 h before training (**A**, **D**), 1 h after training (**B**, **E**); also shown in Figure 2, included here for comparison), or 1 h before testing (**C**, **F**). Panels **A**–**C** show discrimination learning and memory, panels **D**–**F** show aversive learning and memory and panels **G**–**I** show reward learning and memory, during acquisition training (A1,A2,A3) and at the retention test. 1 h before training: *n* PB = 94, *n* vehicle = 93; 1 h after training: *n* PB = 176, *n* vehicle = 184; 1 h before testing *n* PB = 97, *n* vehicle = 95. * *p* < 0.05, *** *p* < 0.001.

Similarly, we examined the effect of PB treatment timing on memory more closely. Bees were treated with PB or vehicle 1 h before training, 1 h after training or 1 h before testing (Figure 4). The results showed that PB treatment 1 h after training very significantly impaired discrimination memory at test (*p* = 0.000), largely due to highly significant impairment of aversive memory (*p* = 0.000) with no effect on reward memory. Treatment with PB 1 h before training or 1 h before testing did not have any significant effect on discrimination, aversive or reward learning or memory (*p* > 0.05). There was a non-significant trend towards improved reward memory at test when bees were treated with PB 1 h before training (*p* = 0.065).

### 3.4. A Range of HDAC Inhibitors Selectively Impair Aversive Memory

**Figure 5:**
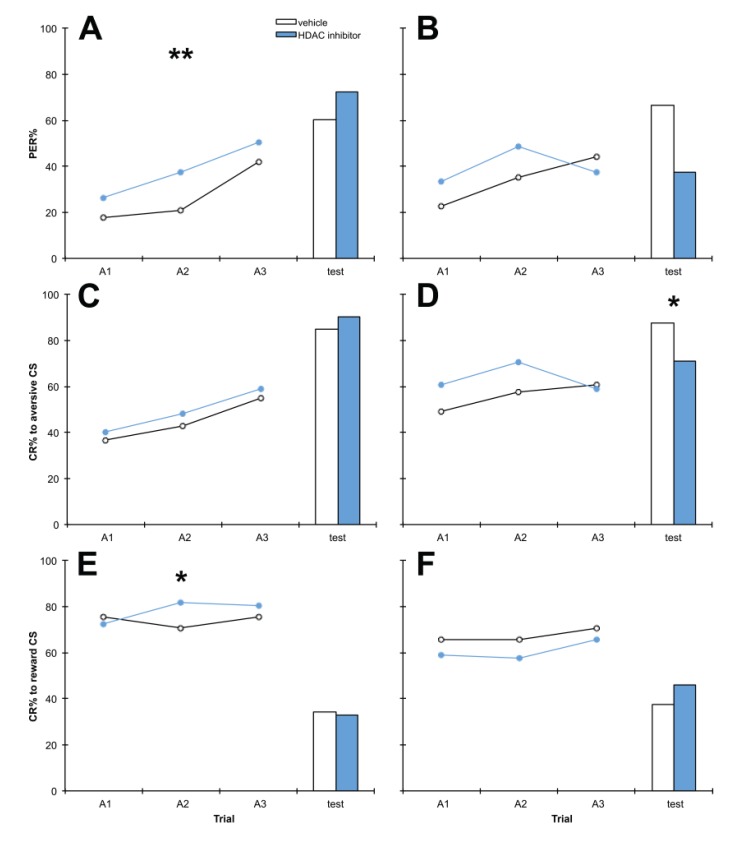
The HDAC inhibitor sodium butyrate (NaB) impairs aversive memory at test at 20 mM but not 10 mM. Bees were treated with 10 mM (**A**, **C**, **E**) or 20 mM (**B**, **D**, **F**) NaB or vehicle only, 1 h before testing. Panels **A** and **B** show discrimination learning and memory, panels **C** and **D** show aversive learning and memory and panels **E**–**F** show reward learning and memory, during acquisition training (A1,A2,A3) and at the retention test. 10 mM: *n* NaB = 115, *n* vehicle = 125; 20 mM: *n* NaB = 52, *n* vehicle = 48. * *p* < 0.05, ** *p* < 0.01.

We also assessed the effects of a third HDAC inhibitor, sodium butyrate (NaB), on aversive and reward memory. Bees were treated with 10 mM or 20 mM NaB, or vehicle only, 1 h before testing (Figure 5). Treatment with 20 mM NaB significantly impaired aversive memory at test (*p* = 0.045), and trended towards impairing discrimination memory though this did not reach statistical significance (*p* = 0.061). Treatment with 10 mM NaB was insufficient to produce this effect on aversive memory (*p* = 0.187). No effects were observed on reward memory at either dose (*p* > 0.05). In the 10 mM NaB experiment, bees in treatment and control groups differed in reward learning at the second training trial, (*p* = 0.024), but no difference was apparent at the first or third training trials. Survival rates of NaB-treated bees were not significantly different from controls at 10 mM or 20 mM (*p* > 0.05). The effects of different treatment times on memory were not assessed for NaB.

### 3.5. HDAC Inhibition Does not Affect Sucrose Sensitivity or Locomotor Activity

**Figure 6:**
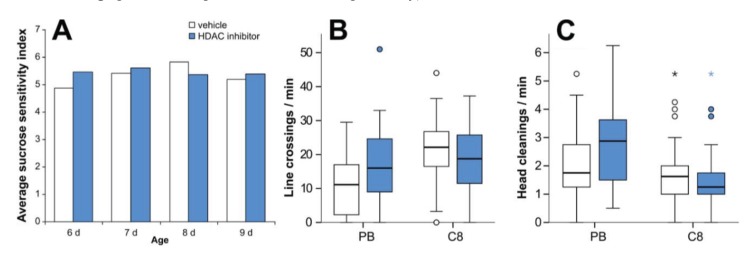
HDAC inhibitor treatment does not affect sucrose sensitivity or locomotor activity. Sucrose sensitivity (**A**): treatment with an HDAC inhibitor does not significantly alter sucrose sensitivity indices at age 6, 7, 8 or 9 d (*p* > 0.05). 10 mM C8 was used to test 6, 8 and 9 d bees, 10 mM PB was used to test 7 d bees. Mean sucrose sensitivity indices are shown. From left to right, *n* HDAC inhibitor = 52, 36, 63 and 40; *n* vehicle = 56, 56, 60 and 51. Locomotor activity (**B**, **C**): bees treated with HDAC inhibitor show no significant change in movement rate (**B**) or grooming behaviour (**C**) relative to controls. By experiment, PB: *n* HDAC inhibitor = 28; *n* vehicle = 28; C8: *n* HDAC inhibitor = 37; *n* vehicle = 38. PB bees were 7 d old, C8 bees were 8 d old. Box plots show medians, quartiles and ranges, with circles and asterisks indicating moderately and strongly outlying data points. No statistically significant effects of PB or C8 treatment were detected on average number of line crossings per minute (*p* = 0.719, 0.254 respectively) or average number of head cleanings per minute (*p* = 0.718, 0.197 respectively).

To determine whether the observed effects of all three HDAC inhibitors on memory could be due to a factor other than memory itself, we assessed the effects of HDAC inhibitor treatment on sucrose sensitivity and locomotion. We tested a range of bee ages, as bees are trained at age 7 d and tested at age 8 d in our protocol, and other drugs have previously been shown to induce precocious PER ability [45]. Our experiments showed that HDAC inhibitor treatment did not significantly affect sucrose sensitivity at any of the ages tested (Figure 6A).

**Figure 7:**
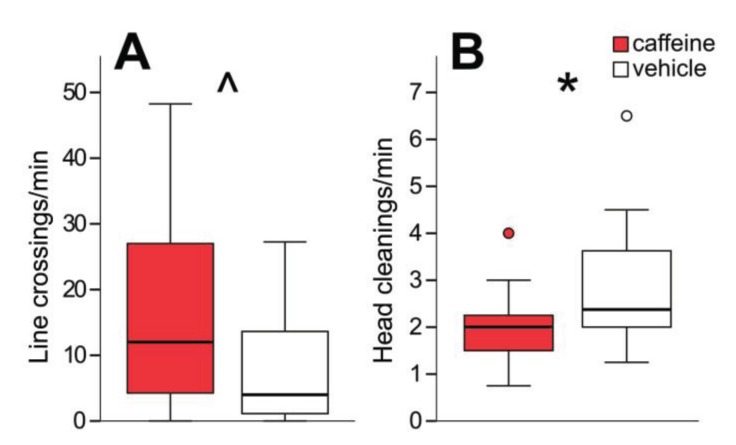
Bees treated with caffeine have increased movement (**A**) and decreased grooming behaviour (**B**). By experiment, 7 d: *n* caffeine = 17, *n* vehicle = 16. Caffeine-treated bees had significantly fewer head cleanings per minute (*p* = 0.045) and showed a trend towards increased line crossings per minute (*p* = 0.074). * *p* < 0.05, **^** *p* < 0.10.

Bees were also assessed for any effects of HDAC inhibition on locomotor activity. Overall movement levels (number of line crossings), and the number of instances of head cleaning (expected to correlate inversely with movement level) were counted. After PB or C8 treatment there were no significant changes to line crossings per minute (*p* = 0.719, *p* = 0.254 respectively) or head cleanings per minute (*p* = 0.718, *p* = 0.197 respectively) (Figure 6B,C). Low doses of caffeine are strong stimulants of locomotor activity [46,47] so the sensitivity of this assay to detect changes in locomotion of the magnitude produced by caffeine treatment was also assessed, as a positive control. Caffeine-treated bees showed a trend towards increased movement levels (*p* = 0.074) and had significantly decreased grooming behaviour (*p* = 0.045; Figure 7). This confirms that the assay used here can detect changes in locomotion at the level affected by a locomotor stimulant, and that no such changes occur following HDAC inhibition. This suggests that the observed effects of HDAC inhibition on aversive and reward memory are truly attributable to memory, rather than altered sucrose sensitivity or locomotion.

## 4. Discussion

The role of histone acetylation in memory is becoming increasingly well characterised across a range of learning paradigms and model animals. Much of what we have learnt from rodent models has made use of aversive learning, yet the role of histone acetylation in aversive memory in the honey bee, a popular invertebrate model for both memory and epigenetics, was previously unknown. The role of histone acetylation in aversive and reward memory in the honey bee is essential knowledge for uncovering the evolution of memory processing, assessing commonalities between vertebrate and invertebrate models, and is of particular interest following the discovery of parallels between DNA methylation in rodent and honey bee memory [4,6].

### 4.1. C8 and PB Impair Aversive Memory but not Reward Memory in a Dose-Dependent Manner

Bees treated with the HDAC inhibitors C8 and PB exhibited significantly impaired performance in discrimination memory tests, due to impairment of aversive memory but not reward memory (Figure 1 and Figure 2). To our knowledge this is the first assessment of the role of histone acetylation in aversive memory in the honey bee, and it uncovered a striking contrast with reward memory, which was unaffected by treatment with HDAC inhibitors. Memory impairment by HDAC inhibition has been found previously in other settings: HDAC inhibition impairs novel object location in the rat [19], and intriguingly, is associated with cognitive impairment in humans [48,49,50,51], but our results are the first demonstration of HDAC inhibition impairing memory in the honey bee. If HDAC inhibition impairs memory then there are worrying implications for the clinical use of HDAC inhibitors, as many (including PB) are currently used therapeutically [52].

HDAC inhibitors alter transcription via manipulation of the chromatin fibre structure, so our demonstration of an effect of two distinct HDAC inhibiting drugs selectively upon aversive memory suggests that different transcriptional pathways may be involved in aversive and reward memory in the honey bee. Reward and aversive memory are mediated by the biogenic amines octopamine and dopamine respectively in insects, including *Drosophila* [53], crickets [54] and the honey bee [24,55,56]. Greater regulation of aversive memory by histone acetylation could therefore be explained by increased vulnerability of transcriptional pathways modulated by dopamine, to increased levels of histone acetylation.

We hypothesise that the impairment of one memory type by HDAC inhibition while another is unaffected could be underpinned by increased histone acetylation upregulating transcription of different memory enhancers and suppressors in the genetic circuitries known to contribute to aversive and reward memory. HDAC inhibitors affect the transcription of only a small and specific subset of genes [57,58], and are thought to improve memory by increasing transcription of genes leading to synaptogenesis and dendritic growth [10,18]. Our results suggest the hypothesis that in the honey bee, HDAC inhibitor treatment increases the expression of genes that suppress aversive memory.

One suggested category of such memory suppressor genes is comprised by protein phosphatases (PPs) that regulate many cellular processes by the reversible phosphorylation of specific substrates. For example, the Ca2+/calmodulin-dependent protein phosphatase PP2B has been shown to block learning, memory storage and memory retrieval, whereas PP1 that acts downstream from calcineurin and negatively regulates synaptic plasticity is involved in the mechanisms that constrain learning efficacy and underlie forgetting. Importantly, inhibition of DNA methylation in a rodent model leads to abnormal transcription of PP1 that interferes with memory formation [59]. Although highly conserved PPs are encoded by the honey bee genome it is not clear if they are involved in memory suppression. However, genes coding for PPs in the honey bee are methylated and some are differentially methylated in brains of queens and workers suggesting that their transcription is modulated by epigenetic mechanism [60]. Indeed, pharmacological inhibition of DNA methylation in honey bee workers brains results in the impairment of extinction memory and interferes with the process of erasing prior memory traces [4].

We did not observe an improvement in reward memory upon C8 or PB treatment, as has been previously reported after treatment with the HDAC inhibitor TSA [21], but the aversive memory impairment we report here is consistent and highly reproducible. This discrepancy is most likely attributable to differences between HDAC inhibitors: HDAC-inhibiting drugs vary in their ability to target different HDAC enzyme isoforms [61,62,63], so TSA cannot be expected to have identical effects to C8 and PB. The involvement of specific HDAC isoforms in memory has been demonstrated in mice: overexpression of HDAC2, but not HDAC1, boosted contextual fear memory [10], and HDAC9 translocation regulates dendritic growth and the activity-dependent expression of immediate-early genes such as *c-fos* [64]. Future experiments should address the effect of TSA on aversive memory in honey bees.

**Figure 8:**
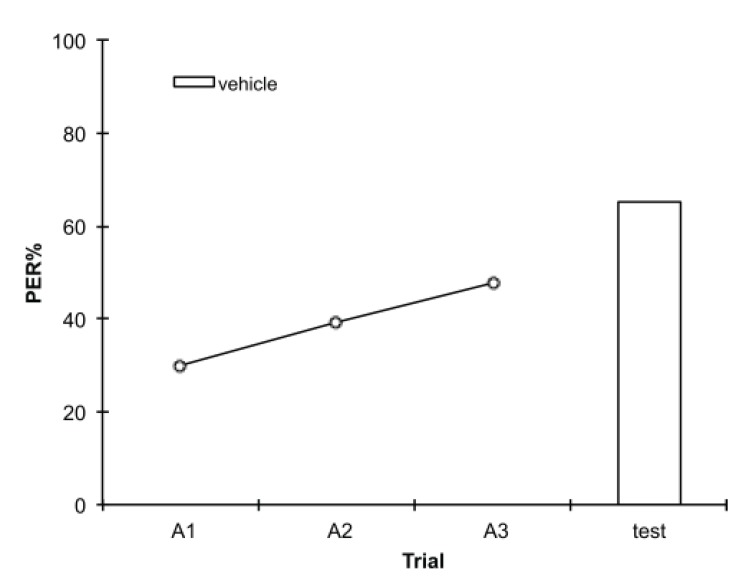
In this version of the proboscis extension reflex (PER) assay, discrimination learning increases linearly during training, and at the 24 h test approximately 2/3 of bees displayed the correct double response, indicating successful discrimination memory. Shown here are control bees from all experiments pooled (*n* = 902).

In this PER paradigm, discrimination learning typically increases during training and reaches around 66% at the 24 h retention test (e.g., [4,32]). We confirmed that this is also the case in the experiments presented here by pooling control group bees across all experiments (Figure 8). This showed a clear linear increase in correct discrimination responses with training, even though this overall trend may not be visible in each experiment due to the lower numbers of bees.

We also examined the dose-dependence of the effects of HDAC inhibitor treatment on memory. Effects were visible after treatment with 10 mM or 20 mM but not 1 mM C8 (Figure 1), suggesting 10 mM C8 is the minimum dose required to produce an effect. PB treatment had effects specifically at 10 mM but not 1 mM or 50 mM (Figure 2), indicating that the effects of PB on aversive memory only occur at moderate doses. Neither C8 nor PB significantly altered survivorship throughout the experiment, providing further evidence that neither HDAC inhibitor is toxic to honey bees at 10 mM. This concentration was therefore selected for both C8 and PB treatments in subsequent experiments. This corresponds to 2.8 μg C8 and 1.9 μg PB per treatment, which for a honey bee weighing 80 mg equates to doses of 35 mg/kg C8 and 24 mg/kg PB. While C8 has been shown to increase H3K9 acetylation in mouse oocytes *in vitro* [65], to our knowledge our experiments constitute the first report of C8 administration to a living organism. In mice, treatment with PB at 200 mg/kg daily for five weeks has been shown to affect memory without toxic effects [37].

### 4.2. Treatment with C8 1 h before Training, 1 h after Training or 1 h before Testing Impairs Aversive Memory but Has no Effect on Reward Memory

Manipulating treatment time relative to the training and testing procedure is a tool used in pharmacological dissection of memory processing to selectively target phases of memory processing [4,66,67,68]. Of course, the ability to selectively target one of these phases depends on the *in vivo* stability of the drug being administered: C8 is a novel synthetic drug and its stability within a living organism remains uncharacterised, to our knowledge. That we see effects of C8 treatment on aversive memory performance at test with all three treatment times (Figure 3D–F) could indicate that this HDAC inhibitor has extensive effects throughout aversive memory processing (formation, consolidation and recall), or, it could be that C8 impairs recall alone and has a long enough half-life to still impair recall when administered prior to training. Future experiments should aim to establish the stability of C8 *in vivo*.

The experiments presented herein show that C8 treatment impairs aversive memory recall, at minimum. In the honey bee histone acetylation is known to be involved in the consolidation phase of reward memory [21] though the effects of HDAC inhibition on reward memory recall were not examined in this study. In rodent model systems histone acetylation is known to be involved in memory formation [11,12] and consolidation [69].

Intriguingly, treatment with C8 1 h before training also significantly enhanced discrimination learning at the third training trial during training (Figure 3A), due to improved aversive learning (Figure 3D), with no change in reward learning (Figure 3G). Improvements to memory upon treatment with HDAC inhibitors have been demonstrated previously in a range of paradigms, as discussed earlier. Considering the significant and reproducible impairment of aversive memory at test, an effect on the same type of memory (aversive) but in the opposite direction firstly supports our hypothesis that aversive memory is more vulnerable to HDAC inhibitors than reward memory, and secondly suggests effects of C8 on two separate mechanisms: one of which selectively improves aversive learning 1 h later, and another which selectively impairs aversive memory recall at test.

It could also be that HDAC inhibiting drugs act on memory independent of transcription [21], certainly a transcription-independent effect would fit with current knowledge of the time course of transcriptional requirements in memory in the honey bee [4,68,70]. Drugs administered topically to the honey bee thorax reach the head within 15 min, and an equal or slightly greater proportion of the drug reaches the head within 1 h of thoracic topical application than thoracic injection [41]. Thoracic injection of TSA and Garcinol alter histone acetylation levels in the brain 2 h after treatment [21]. If C8 and PB are transported similarly then it is reasonable that effects on memory observed 1 h after treatment could be due to HDAC inhibitors acting within the brain to alter memory.

### 4.3. Treatment with PB 1 h after Training Impairs Aversive Memory but Has no Effect on Reward Memory

We also examined the effects of PB treatment 1 h before training, 1 h after training or 1 h before testing on aversive and reward memory, similar to the previous experiment. In contrast to the broad effects of C8, PB only impaired aversive memory at test when administered 1 h after training (Figure 4D–F). The speed with which drugs topically applied to the thorax reach the honey bee head, in conjunction with the relatively short half-life of PB—in primates, PB concentration peaks 40 min after treatment in plasma and 80 min after treatment in cerebrospinal fluid [63]—are expected to provide a relatively narrow window of effectiveness in the honey bee. Our results suggest that PB treatment 1 h after training affects a critical time period for aversive memory processing. Most likely, PB is impairing consolidation of the aversive memory in the hours following training, which then produces impaired aversive memory at the test 24 h after training (Figure 4E). A role for histone acetylation in consolidation of aversive memories would parallel the effect of the HDAC inhibitor TSA on consolidation of reward memories in the honey bee [21].

### 4.4. A Range of HDAC Inhibitors Selectively Impair Aversive Memory

The HDAC inhibitors C8 and PB both impaired discrimination memory, by impairing aversive memory while at the same time leaving reward memory unaffected. This selective impairment of aversive memory was robust, highly reproducible and dose-dependent (Figure 1, Figure 2, Figure 3 and Figure 4). Since different HDAC inhibitors target different isoforms of the HDAC enzyme [61,62,63], we also examined whether a third HDAC inhibiting drug, sodium butyrate (NaB) selectively impaired aversive memory. We hypothesised that other HDAC inhibiting drugs should also selectively impair aversive memory, if this effect is due to inhibition of HDAC enzymes. Indeed, our results showed that treatment with 20 mM NaB impaired aversive memory at test, with no effect on reward memory (Figure 5C–F). There was a trend towards impaired discrimination memory at test resulting from this impaired aversive memory (*p* = 0.061, Figure 5B), however this did not reach statistical significance, likely due to the number of subjects. This experiment has therefore reproduced the selective impairment of aversive memory, the main effect reported here in our pharmacological dissection of memory, and extended this to a third HDAC inhibiting drug.

### 4.5. HDAC Inhibition Does not Affect Locomotor Activity or Sucrose Sensitivity

Impaired aversive memory corresponds to more frequent extension of the proboscis by HDAC inhibitor-treated bees which, even though reward memory was unaffected, could indicate an effect of these drugs on sucrose sensitivity or locomotor activity rather than memory itself. Higher responsiveness to sucrose increases PER learning [71]. However, our results show no effect of HDAC inhibitors on sucrose sensitivity index, a marker of the value of the reinforcing stimulus, or locomotor activity levels (Figure 6). This therefore suggests that the observed effects of HDAC inhibitors on aversive memory are indeed more likely due to *bona fide* interference with memory rather than with a non-memory factor such as sucrose sensitivity or locomotion.

Similarly, the HDAC inhibitor TSA does not affect sucrose responsiveness, sensitisation or habituation in the honey bee [21], and in the rat freezing behaviour itself is not altered by NaB treatment, nor does NaB injection cause any general malaise or fear [11]. Chronic NaB, SAHA or VPA treatment also has no effect on exploratory activity or post-shock freezing in mice [61]. That aversive memories were selectively inhibited while reward memories remained unaffected by HDAC inhibition, supports the notion that these are genuine and specific effects on aversive memory–if the effects of HDAC inhibition were non-specific, reward memory would be expected to be affected too. The notion of a specific role for histone acetylation in aversive memory is further strengthened by three separate drugs that all inhibit HDACs impaired aversive memory. However, one caveat on all research utilising HDAC inhibitors is that HDAC enzymes do deacetylate other proteins as well as histones [62]. Also, it could be argued that since our version of the PER assay does not employ a control odour to test for generalisation of the CR to novel odours [72], it is possible that the impairment to aversive memory could represent increased proboscis extension to any odour other than the reward CS. This is possible, though generalisation is typically only to chemically similar odours [73], and in our assay there were no other odours present as an extractor fan was utilised throughout PER assays.

## 5. Conclusions

Here we report that histone acetylation is extensively involved in aversive memory in the honey bee. Treatment with HDAC inhibiting drugs C8, PB and NaB impaired aversive memory while leaving reward memory unaffected, presumably via regulation of histone acetylation levels. Selective impairment of aversive memory was robust, reproducible and dose-dependent. Treatment with PB 1 h after training impaired memory at test, suggesting specific effects on memory consolidation, whereas treatment with C8 at any of the treatment times tested was sufficient to impair aversive memory, indicating either broader effects of this drug on memory processing or a considerably longer half-life of C8 than PB *in vivo*. Impaired aversive memory was also observed following treatment with a third HDAC inhibiting drug, NaB, supporting the robustness of this effect. The effects of HDAC inhibitors on performance in the PER assay are likely to be *bona fide* effects on memory processing as HDAC inhibition had no effect on sucrose sensitivity or locomotion. That we tested and saw similar effects for three distinct HDAC inhibiting drugs is strength of this study. The conservation of a role for histone acetylation in aversive memory between vertebrates and the honey bee parallels the conservation of DNA methylation, and expands our knowledge of epigenetic control of neural plasticity in the honey bee. In a broader context, our findings illustrate the universality of the epigenetic code in generating behavioural complexities from a fixed genetic hardware [74].
